# Reduced health-related quality of life in children born extremely preterm in 2006 compared with 1995: the EPICure Studies

**DOI:** 10.1136/archdischild-2021-322888

**Published:** 2021-10-25

**Authors:** Yanyan Ni, Samantha Johnson, Neil Marlow, Dieter Wolke

**Affiliations:** 1 Institute for Women's Health, University College London, London, UK; 2 Department of Psychology, University of Warwick, Coventry, UK; 3 Department of Health Sciences, University of Leicester, Leicester, UK; 4 Division of Health Sciences, University of Warwick, Warwick Medical School, Coventry, UK

**Keywords:** neonatology, child health

## Abstract

**Objective:**

To compare health-related quality of life (HRQL) in childhood for extremely preterm (EP) births before 26 weeks of gestation in England in two eras: 1995 and 2006.

**Design:**

Prospective cohort studies.

**Setting:**

School or home-based assessments at 11 years of age.

**Participants:**

Available data for 88 EP children born before 26 weeks of gestation in 2006 (EPICure2) were compared with those of 140 born in England during 1995 (EPICure). To account for social secular trends, the comparison between eras was also made for term-born controls as reference.

**Main outcome measures:**

HRQL was measured using the parent-completed Health Utilities Index (HUI) questionnaire with utility scores calculated using the HUI3 classification system. Eight attributes were assessed: vision, hearing, speech, ambulation, dexterity, emotion, cognition and pain.

**Results:**

At 11 years, mean utility scores were significantly lower in EPICure2 (2006) than in EPICure (1995; Δ −0.12, 95% CI −0.20 to –0.04). The difference increased (Δ −0.27, 95% CI −0.41 to –0.12) after adjusting for significant perinatal and demographic differences between cohorts. Rates of suboptimal function were increased in EPICure2 for all eight attributes, but statistically significant differences were only found in speech (p=0.004) and dexterity (p=0.020). After excluding children with severe neurodevelopmental impairment, the adjusted difference between cohorts remained significant but attenuated (−0.14 (−0.26 to –0.01)). Mean utility scores for controls were similar between cohorts (Δ −0.01 (−0.04 to 0.02)).

**Conclusions:**

Using parent report, there was a clinically significant decline in HRQL ratings for EP children over time. Areas contributing the most to the decline were speech and dexterity.

**Trial registration number:**

ISRCTN86323684.

## Abstract (precis)

Parent-reported health-related quality of life information was obtained for children born <26 weeks gestation in England at 11 years in two birth cohorts (1995 and 2006). Mean utility scores were significantly lower for 2006 vs. 1995 cohorts, while control groups were similar.

**What is already known on this topic?**

- Health-related quality of life (HRQL) reflects the impact of health on one’s overall emotional, social and physical well-being.
- School-aged children, adolescents and young adults born extremely preterm (EP) have lower HRQL than term-born peers.
- Little is known about whether HRQL in EP children has changed over time with changed neonatal treatment approaches.

**What this study adds?**

- Using parent report, there was a clinically important decline in HRQL at 11 years of age for children born extremely preterm between 1995 and 2006.
- There were reductions in all eight attributes of HRQL, but only those for speech and dexterity were significant.
- Interventions with a focus to improve speech and motor skills, together with long-term support for parents, might help to optimise long-term outcomes.

## Introduction

Survival for babies born extremely preterm (EP) has improved since 1995 in England^1 2^ and in other European countries.^3–5^ We previously reported that improved survival was not paralleled by improved head growth or improved long-term neurodevelopmental and educational outcomes.^6 7^ Apart from these objectively assessed indicators, it has become increasingly important to understand health-related quality of life (HRQL) of EP children. According to the WHO, quality of life is defined as an individual’s perception of their position in life in the context of the culture and value systems in which they live and in relation to their goals, expectations, standards and concerns.^8^ HRQL assesses children’s overall emotional, social and physical well-being. Using both self-reports and parent reports, school-aged children and adolescents born EP were found to have lower HRQL than term-born peers.^9–15^ However, little is known as to whether HRQL in EP children born in different eras of obstetric and neonatal care has changed over time and improved in line with increased survival.

Recent evidence from the Victorian Infant Collaborative Study (VICS) Group suggests worsening HRQL at 8 years of age in children born <28 weeks of gestation across three eras (1991–1992, 1997 and 2005 cohorts).^16^ Peart *et al* 16 used the Health Utilities Index (HUI) questionnaire completed by parents to measure HRQL with overall multi-attribute utility (MAU) scores calculated using a published utility algorithm based on community preferences.^17^ A major limitation with this study is the use of two different HUI classification systems in the different cohorts: the HUI2 system in the 1991–1992 and 1997 cohorts and the HUI3 system in the 2005 cohort. HUI2 covers seven basic attributes: sensation, mobility, emotion, cognition, self-care, pain and fertility, each attribute with three to five levels. HUI3 covers eight attributes: vision, hearing, speech, ambulation, dexterity, emotion, cognition and pain, each graded on a 5-point or 6-point scale corresponding to the level of severity, ranging from normal function (level 1) to severe impairment (levels 5–6).^18 19^ Research shows that HUI2 MAU scores are systematically higher than HUI3 scores.^20^ Therefore, it is difficult to determine whether the findings in VICS reflect a true decline in HRQL in the 2005 cohort compared with earlier eras or whether it is due to the use of different HUI systems. Furthermore, their study did not assess the difference in individual attributes, thus it is impossible to know which areas may contribute most to the reduction in overall HRQL.

The HUI3 classification system has been shown to be reliable, responsive and valid,^19^ and has been frequently used in EP populations.^12 15 21 22^ In this study, we investigate whether HRQL in children born EP improved, remained unchanged or declined over time by comparing the HUI3 MAU scores at 11 years of age in EP children (<26 weeks of gestation) born in 1995 and in 2006 in England. If there was a significant change, we would further explore which attributes show significant change.

## Methods

### Study design and participants

The EPICure2 Study comprised all EP births <27 weeks of gestation in England during 2006.^1^ Of 1031 babies who survived to 3 years of age, invitations to participate in the 11-year follow-up were sent to a sample of parents of 482 children comprising births in 17 clinical neonatal networks in England. As part of the study design, a contemporary comparison group of term-born children was recruited, which has been described in detail previously.^6^ In total, 200 EP children and 143 term-born controls were assessed at age 11 years. The EPICure Study comprised all births <26 weeks of gestation in the UK and Ireland from March to December 1995. Recruitment of the cohort and controls has been described previously.^23 24^ To compare HRQL between the EPICure and EPICure2 cohorts, we restricted participants to EP children born <26 weeks of gestation to women residing in England.

### Measures

In both cohorts, HRQL was assessed by parent report using the 15-item HUI questionnaire. The level of function within each attribute is graded on a 5-point or 6-point scale, ranging from normal function (level 1) to severe impairment (levels 5–6).^25^ Responses were mapped onto the HUI3 classification system. An overall MAU score was calculated for each participant using a published utility algorithm based on preferences of a randomly selected general population sample of Canadian adults.^17^ MAU scores in this study indicate children’s HRQL based on societal standards ranging from −0.36 (worst state) to 1.00 (perfect health) on an interval scale. Function within each attribute was recorded as suboptimal if any level of functional impairment (level 2 or above) was reported.^26 27^ The number of single attributes that were suboptimal was then calculated.

Data on neurodevelopmental impairment at 11 years were collected in both cohorts. Severe neurodevelopmental impairment was defined as one or more of the following^6 28^: cognitive impairment (classified as a score >3 SDs below the mean of controls using the Kaufman-Assessment Battery for Children (EPICure: first edition; EPICure2: second edition)), blindness, profound hearing loss or cerebral palsy (the Gross Motor Function Classification System (GMFCS) or the Manual Abilities Classification System levels 3–5). Similar definitions were used for severe impairment at 2.5/3 years of age in both cohorts^2^: any of cerebral palsy (GMFCS levels 3–5), blindness, profound sensorineural hearing loss not improved by aids or a developmental quotient less than 3 SDs below the mean for age.

Perinatal and demographic variables that were available in both cohorts included gestational age in weeks, birth weight (grams), participant sex assigned at birth (male vs female), multiple birth, breast milk at any time (yes vs no), enteral feeding begun before day 7 (yes vs no), antenatal systemic steroids (yes vs no), postnatal systemic steroids (yes vs no), ethnicity (white, Asian, black or other), maternal education, socioeconomic status and age at the 11-year assessment. Maternal education was collected at 11 years using parent questionnaires and classified using the International Standard Classification of Education (ISCED)^29^: (1) low level: equivalent to ISCED 0–2; (2) medium: ISCED 3–5; (3) high: ISCED 6–8. Missing values for children born <26 weeks of gestation in England (>20% missing data in both cohorts) were imputed using data collected at 2.5 and 6 years of age. The Index of Multiple Deprivation 2015 (IMD), the version closest to assessment dates, was used as a measure of socioeconomic status at 11 years in EPICure2 and was obtained using postcode of parents’ residence at the time of the assessment.^30^ IMD ranks were used to derive deciles based on the English population with decile 1 (most deprived) to decile 10 (least deprived). The IMD 2007 version was used at the 11-year assessment in EPICure.

### Data analysis

**Figure 1:**
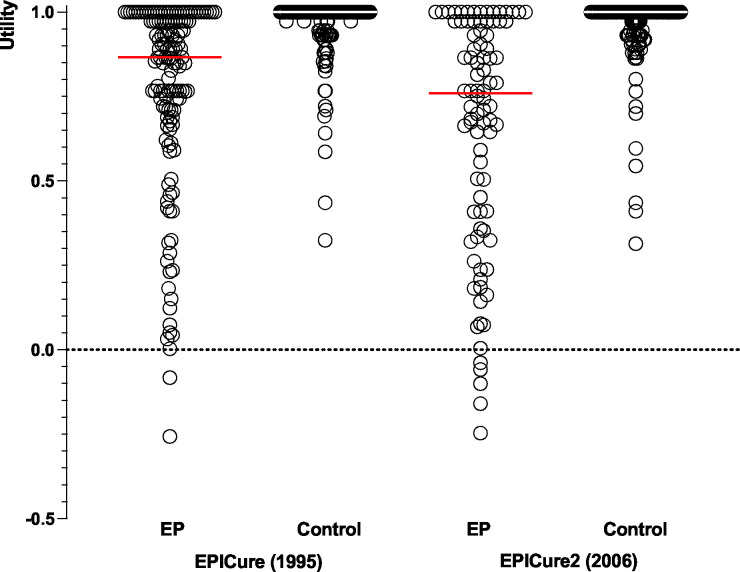
Distribution of the HUI3 utility scores for children born <26 weeks of gestation in England and term-born controls. EP, extremely preterm; HUI, Health Utilities Index.

Analyses were performed in STATA V.16.1. Descriptive statistics (means and medians) of MAU scores were calculated for EPICure and EPICure2. Score differences between the two cohorts were analysed using linear regression. Perinatal and demographic differences between cohorts were then adjusted for in the regression models. Differences in mean scores (Δ) and their 95% CIs were reported. Because MAU scores were not normally distributed (figure 1), non-parametric analyses (median regression) were conducted to assess the robustness of results obtained using parametric methods. The level of statistical significance was set at <0.05. A score difference of 0.03 or more is regarded as clinically important.^19^ Differences in the rates of suboptimal function within attributes between cohorts were investigated using Χ^2^ tests, while differences in the number of suboptimal attributes were investigated using Wilcoxon rank-sum tests.

## Results

### Attrition and missing data

In EPICure2, 584 children born <26 weeks’ gestation survived to 3 years and 112 were assessed at age 11 years (online supplemental figure 1). Among those assessed, 88 children had complete HUI data and 5 children had partial HUI data. Perinatal and demographic information was compared between children with and without complete HUI data at age 11 years in both cohorts (table 1). In EPICure, 309 children survived to 2.5 years among which 260 were born to mothers residing in England. Of 176 assessed children at age 11 years, 140 had complete HUI data and 11 children had partial HUI data. In EPICure2, children with HUI data were more likely to be born to mothers of older age and to have received breast milk at discharge. There were no significant differences between those with and without HUI data in gestational age, birth weight and other birth characteristics (eg, enteral feeding, the use of antenatal or postnatal steroids). In EPICure, children with HUI data were significantly more likely to be from a multiple birth, to have mothers of higher education, and to have higher IQ scores and a lower proportion of severe neurodevelopment impairment at 2.5 years.

10.1136/fetalneonatal-2021-322888.supp1 Supplementary data

**Table 1:** Drop-out and missing data analysis in EPICure and EPICure2

		EPICure 1995		EPICure2 2006		P value (a) vs (b)	P value (c) vs (d)	P value (b) vs (d)
		2.5-year sample without complete HUI data at 11 years* (a)	2.5-year sample with complete HUI data at 11 years (b)	3-year sample without complete HUI data at 11 years* (c)	3-year sample with complete HUI data at 11 years (d)			
		n=120	n=140	n=496	n=88			
**Perinatal variables**								
Gestational age								
<24 weeks	% (n/N)	8.3 (10/120)	10.0 (14/140)	11.3 (56/496)	11.4 (10/88)	0.895	0.342	0.467
24 weeks	% (n/N)	32.5 (39/120)	31.4 (44/140)	31.5 (156/496)	23.9 (21/88)			
25 weeks	% (n/N)	59.2 (71/120)	58.6 (82/140)	57.3 (284/496)	64.8 (57/88)			
Birth weight (g)	Mean (SD)	739.7 (112.8) (n=120)	755.4 (107.3) (n=140)	732.6 (120.6) (n=496)	750.2 (118.2) (n=88)	0.252	0.206	0.733
Multiple birth	% (n/N)	19.2 (23/120)	31.4 (44/140)	21.8 (108/496)	23.9 (21/88)	0.024	0.663	0.218
Maternal age at delivery	Mean (SD)	28.1 (6.2) (n=120)	28.8 (5.7) (n=139)	29.1 (6.4) (n=496)	30.8 (6.1) (n=88)	0.337	0.020	0.013
Breast milk at any time	% (n/N)	80.8 (97/120)	88.6 (124/140)	95.4 (472/495)	100.0 (88/88)	0.082	0.039	0.001
Enteral feeding begun before day 7	% (n/N)	43.7 (52/119)	51.1 (69/135)	81.7 (405/496)	85.2 (75/88)	0.238	0.419	<0.001
Antenatal systemic steroids	% (n/N)	76.7 (92/120)	82.7 (115/139)	87.4 (429/491)	90.9 (80/88)	0.224	0.349	0.085
Postnatal systemic steroids	% (n/N)	73.3 (88/120)	69.8 (97/139)	19.6 (97/496)	25.0 (22/88)	0.528	0.243	<0.001
**Demographic variables**								
Age assessed at 11 years	Mean (SD)	–	10.8 (0.3) (n=140)	–	11.8 (0.5) (n=88)	–	–	<0.001
Male sex	% (n/N)	55.0 (66/120)	43.6 (61/140)	47.2 (234/496)	52.3 (46/88)	0.066	0.378	0.200
Ethnicity								
White	% (n/N)	66.7 (80/120)	79.1 (110/139)	63.3 (311/491)	60.5 (52/86)	0.051	0.290	0.002
Asian	% (n/N)	10.8 (13/120)	4.3 (6/139)	9.6 (47/491)	16.3 (14/86)			
Black	% (n/N)	19.2 (23/120)	15.8 (22/139)	22.6 (111/491)	18.6 (16/86)			
Other	% (n/N)	3.3 (4/120)	0.7 (1/139)	4.5 (22/491)	4.7 (4/86)			
Maternal education								
Low	% (n/N)	25.5 (24/94)	10.1 (14/138)	9.0 (21/233)	4.9 (4/82)	0.007	0.111	<0.001
Medium	% (n/N)	61.7 (58/94)	76.8 (106/138)	60.1 (140/233)	52.4 (43/82)			
High	% (n/N)	12.8 (12/94)	13.0 (18/138)	30.9 (72/233)	42.7 (35/82)			
IMD at 11 years	Mean (SD)	–	5.3 (2.8) (n=138)	–	5.2 (2.8) (n=87)	–	–	0.814
**Developmental variables**								
IQ score at 2.5/3 years	Mean (SD)	76.1 (14.7) (n=75)	83.6 (13.3) (n=132)	86.4 (20.1) (n=250)	85.5 (19.1) (n=75)	<0.001	0.719	0.405
Severe neurodevelopmental impairment at 2.5/3 years	% (n/N)	32.3 (31/96)	15.8 (22/139)	16.8 (42/250)	14.7 (11/75)	0.003	0.661	0.823
IQ score at 11 years	Mean (SD)	–	85.8 (16.4) (n=138)	–	82.2 (19.9) (n=88)	–	–	0.142
Severe neurodevelopmental impairment at 11 years	% (n/N)	–	7.9 (11/140)	–	5.7 (5/88)	–	–	0.327

*This could be due to drop out, non-completion of parent questionnaires or missing values in one or more of the eight attributes required to compute the utility score.

HUI, Health Utilities Index; IMD, index of Multiple Deprivation.

### Comparative characteristics between cohorts

Compared with EPICure, EPICure2 children were more likely to have received breast milk, to have enteral feeding begun earlier, and to have mothers of older age and with higher educational attainment (table 1); they were less likely to have received postnatal systemic steroids; there were more Asian and black children and fewer white children in EPICure2. At the 11-year assessment, children’s chronological ages were significantly higher in EPICure2. The two cohorts were evenly matched in birth weight, gestational age, sex, multiple birth, IQ scores and rates of severe impairment at age 2.5/3 years, as well as IQ scores, rates of severe impairment and IMD at age 11 years. Differences in perinatal and demographic characteristics were accounted for when comparing HRQL between cohorts.

### Comparison of HRQL between cohorts

At 11 years, using parent report, children in the EPICure2 cohort had significantly lower mean MAU scores than children in the EPICure cohort (0.65 vs 0.77; Δ −0.12, 95% CI −0.20 to –0.04). After adjusting for significant perinatal and demographic differences between cohorts, the difference in mean scores increased (Δ −0.27, 95% CI −0.41 to –0.12). Similar results were shown using median regression (unadjusted Δ −0.11, 95% CI −0.21 to –0.02; adjusted Δ −0.25, 95% CI −0.46 to –0.04). After removing children with severe impairment, the difference between cohorts remained significant but attenuated (table 2). Mean MAU scores for term-born controls were similar between cohorts (table 2; Δ −0.01, 95% CI −0.04 to 0.02).

**Table 2:** A comparison of HUI3 multi-attribute utility scores at 11 years of age for children born <26 weeks of gestation in England and term-born controls in 1995 (EPICure) and 2006 (EPICure2)

	EPICure 1995		EPICure2 2006		Difference in means (95% CI) 2006 vs 1995	
	Mean (SD)	Median (IQR)	Mean (SD)	Median (IQR)	Unadjusted	Adjusted*
EP children	0.77 (0.28) (n=140)	0.87 (0.70–0.97)	0.65 (0.35) (n=88)	0.76 (0.38–0.97)	−**0.12 (−0.20 to –0.04**)	−**0.27 (−0.41 to –0.12**)
EP children after excluding those with severe impairment	0.83 (0.21) (n=122)	0.91 (0.77–1.00)	0.75 (0.28) (n=66)	0.86 (0.65–0.97)	−**0.08 (−0.15 to –0.01**)	−**0.14 (−0.26 to –0.01**)
Controls	0.96 (0.10) (n=141)	1.00 (0.95–1.00)	0.95 (0.12) (n=120)	1.00 (0.93–1.00)	−0.01 (−0.04 to 0.02)	–

Bold font indicates significant changes over time.

*Adjusted for significant differences between the two cohorts, including ethnicity (white, black, South Asian or other), maternal education (low, medium or high), maternal age at birth, any breast milk (yes or no), enteral feeding by day 7 (yes vs no), postnatal systemic steroids (yes vs no) and exact age at the 11-year assessment.

EP, extremely preterm; HUI, Health Utilities Index.

**Figure 2:**
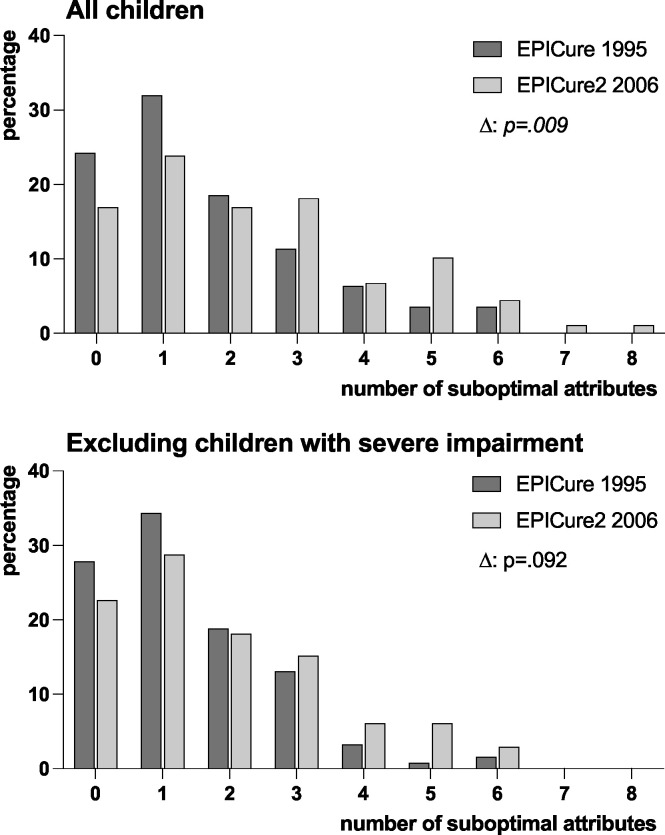
The number of single attributes that were suboptimal at age 11 years for children born <26 weeks of gestation in England in 1995 (EPICure) and 2006 (EPICure2).

Children in the EPICure2 cohort had higher rates of suboptimal function in all eight attributes than children in the EPICure cohort (table 3), but significant differences were only found in speech (p=0.004) and dexterity (p=0.020). Results were similar when excluding children with severe impairment, but the significant difference was only in dexterity (p=0.031). Compared with EPICure, children in the EPICure2 cohort had significantly more suboptimal attributes (figure 2) but after excluding children with severe impairment, this was not significant (p=0.092).

**Table 3:** A comparison of suboptimal function in the HUI3 attributes at age 11 years for children born <26 weeks of gestation in England

HUI3 attributes	EPICure 1995	EPICure2 2006	2006 vs 1995
	% (n/N)	% (n/N)	P value*
EP children			
Vision	32.0 (48/150)	38.5 (35/91)	0.306
Hearing	7.5 (11/147)	12.9 (12/93)	0.165
Speech	21.5 (32/149)	38.7 (36/93)	0.004
Ambulation	10.1 (15/149)	15.2 (14/92)	0.233
Dexterity	11.4 (17/149)	22.6 (21/93)	0.020
Emotion	19.2 (29/151)	22.6 (21/93)	0.526
Cognition	53.0 (79/149)	61.5 (56/91)	0.197
Pain	22.8 (34/149)	28.0 (26/93)	0.368
EP children after removing those with severe impairment			
Vision	28.7 (37/129)	33.8 (23/68)	0.456
Hearing	7.1 (9/127)	10.1 (7/69)	0.455
Speech	15.5 (20/129)	21.7 (15/69)	0.273
Ambulation	3.1 (4/128)	8.7 (6/69)	0.089
Dexterity	5.5 (7/128)	14.5 (10/69)	0.031
Emotion	17.7 (23/130)	23.2 (16/69)	0.353
Cognition	48.4 (62/128)	50.7 (34/67)	0.759
Pain	19.2 (25/130)	24.6 (17/69)	0.374

*P values from the Χ^2^ test.

EP, extremely preterm; HUI, Health Utilities Index.

## Discussion

Using parent report, we showed a decline in HRQL at 11 years of age for children born EP in 2006 compared with those born in 1995. The magnitude of this decline was large, considered as clinically relevant even when those with severe impairment were excluded. There were reductions in all eight individual attribute ratings, but only differences for speech and dexterity were significant. Results were similar after excluding EP children with severe impairment but with a smaller magnitude. In contrast, we found no significant change in HRQL for controls born in 1995 and 2006. Thus, any changes in HRQL ratings of EP children cannot be accounted for by general societal trends.

The use of HUI3 allows comparison of health status across a diverse range of disease areas in child health.^18^ Our results suggest that HRQL of those born EP is perceived by parents as lower than in survivors of childhood cancer (mean utility score range 0.83–0.90).^31^ The reduction in HRQL ratings over time for EP children in our study is consistent with the finding of Peart *et al*.^16^ In their paper, this decline could be explained by poorer academic, motor, and executive functioning in the 2005 cohort compared with the 1991–1992 and 1997 cohorts. However, for our data, we did not find deterioration in neurodevelopmental (cognition, motor) or educational outcomes among children born in the two EPICure cohorts.^6^ We considered that the decline in HRQL ratings could be due to increased difficulties in access to special educational needs (SEN) support. However, based on our data, the proportions of EP children receiving SEN support were similar in both cohorts (EPICure2 53.6% vs EPICure 58.0%; p=0.552). There is little indication of a reduction in educational support for children with academic or behavioural needs between the two eras. Therefore, we speculate on other possible explanations.

Our further analysis showed that areas significantly contributing to the decline were speech and dexterity. In the HUI questionnaire, parents were asked to rate their child’s usual ability to be understood when speaking his/her own language with strangers or with people who know him/her well (speech attribute) and their child’s usual ability to walk around the neighbourhood (dexterity attribute). According to the WHO definition of quality of life, parent reports reflect their perceptions of the health status of their child in relation to their goals, expectations, standards and concerns. Thus, the significant reduction in parental ratings of speech and dexterity might reflect increasing concerns or awareness for their child’s language or social skills and peer relationships, and participation in the community. Language skills and motor abilities are found to be important for developing social skills and forming friendship and peer relationships in childhood.^32 33^ Being born very or extremely preterm has been found to be associated with social withdrawal and poorer social competence,^34^ in addition to increased social exclusion and peer bullying and subsequent mental health problems.^35 36^

Other potential explanations for the reduction in HRQL are increased parental expectations of the health status of preterm-born children or increased parental distress over generations owing to increased social pressure or educational demands, or a tougher work–life balance. There is evidence suggesting that parents who are more distressed report more negative perceptions of their child’s HRQL compared with those less distressed.^37^ Although general societal pressures apply to parents of both EP and control groups, it may be that parents of EP children particularly experience continuous stress and pressure for their children to do well or worry more about their children’s development and outcomes.

The strengths of this paper are the comparison of HRQL from two prospective cohorts recruited 11 years apart, using the same measurement and the same HUI classification system, and adjusting for perinatal and demographic differences in the analysis. Recruitment of a contemporary comparison group of term-born children controlled for general societal changes that may be associated with HRQL. The major limitation relates to drop-out and missing data in both cohorts. Reassuringly, EPICure2 children assessed at 11 years were overall representative of the original cohort in perinatal and demographic characteristics and developmental outcomes at 3 years of age. In EPICure, drop-out and missing data were associated with neurodevelopmental impairment and social disadvantage. However, as reported previously,^15^ mean MAU scores were similar (0.74–0.75) after using multiple imputation. Another limitation is that we only collected HUI data from parents rather than children themselves. A replication using self-report may be required. Adolescents are likely to be the best judge of their own HRQL, especially in attributes relating to emotional and social functioning,^37^ while parental assessment is likely to be affected by their own well-being, expectations and concerns.^37 38^ Finally, HUI3 is an indirect measure of HRQL based on references from a general population of Canadian adults. A replication using the direct approach reflecting individual preferences may be required.

Despite major advances in survival of EP children born between 1995 and 2006, there was a clinically important reduction in HRQL ratings over time. Interventions with a focus to improve speech and motor skills, together with long-term support for parents, might help to optimise long-term outcomes.

## Data Availability

Data are available upon reasonable request. Data are available subject to the EPICure Data Sharing Policy (www.epicure.ac.uk) and will be available as part of the RECAP preterm Cohort Platform (https://recap-preterm.eu).
