# Supplementary material for: Reduced health-related quality of life in children born extremely preterm in 2006 compared with 1995: the EPICure Studies
Source: Arch Dis Child Fetal Neonatal Ed. 2021 Oct 25;107(4):408–13. doi: 10.1136/archdischild-2021-322888 (PMC9209681; doi:10.1136/archdischild-2021-322888)

Figure S1 A flow chart of the two samples of extremely preterm children at 11 years of age born in England in 1995 and 2006.

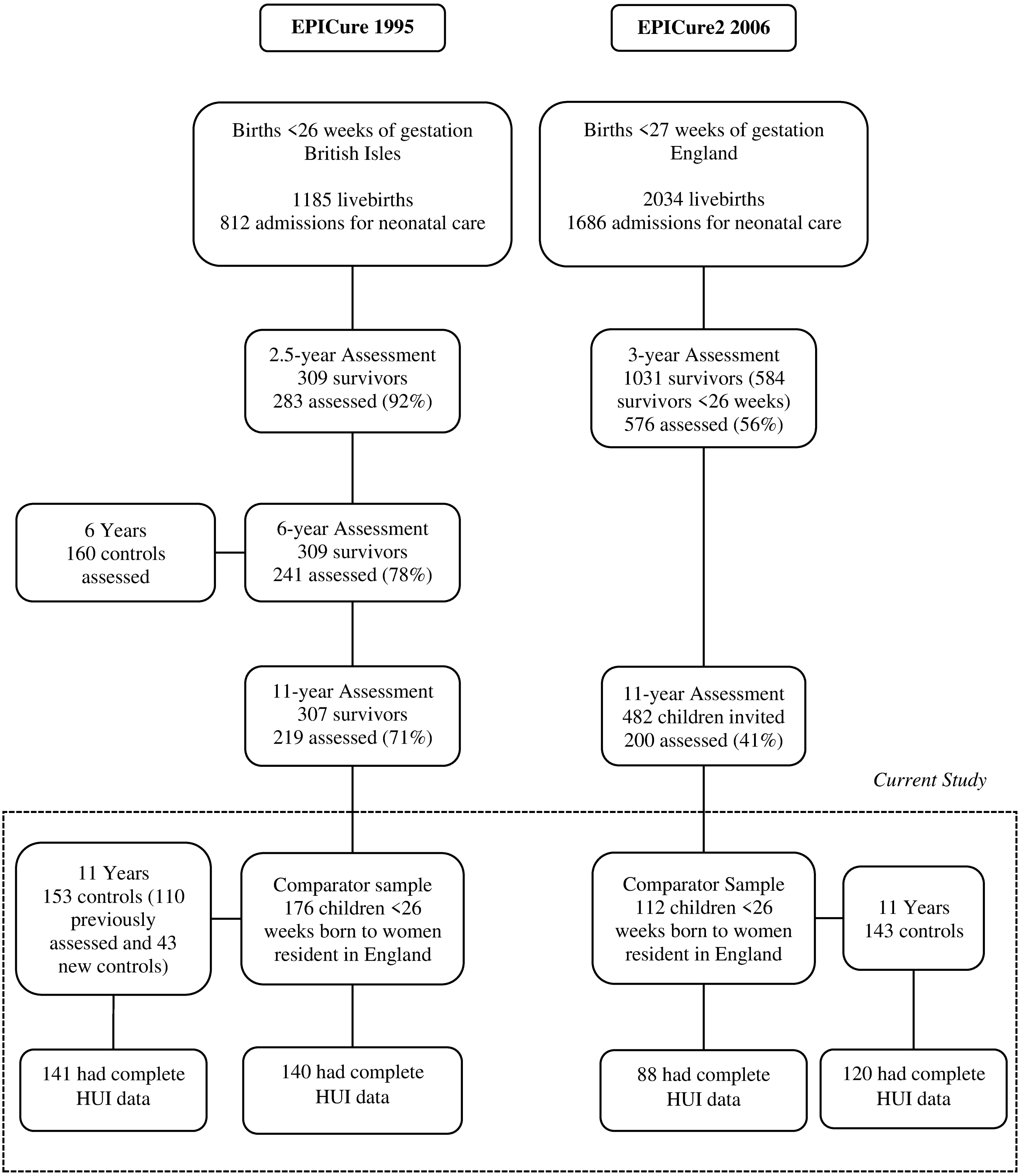

Supplement: Supplementary data [file fetalneonatal-2021-322888supp001.pdf]
